# ErbB2-driven downregulation of the transcription factor Irf6 in breast epithelial cells is required for their 3D growth

**DOI:** 10.1186/s13058-018-1080-1

**Published:** 2018-12-13

**Authors:** Iman Aftab Khan, Byong Hoon Yoo, Michael McPhee, Olivier Masson, Alexi Surette, Kelly Dakin-Hache, Tallal Younis, Gillian Bethune, Kirill V. Rosen

**Affiliations:** 1 Department of Pediatrics, Dalhousie University, Halifax, NS Canada; 2 Department of Biochemistry and Molecular Biology, Dalhousie University, Halifax, NS Canada; 3 Department of Pathology, Dalhousie University, Halifax, NS Canada; 4 Department of Medicine, Dalhousie University, Halifax, NS Canada; 5 Atlantic Research Centre, Rm C-304, CRC, 5849 University Avenue, PO Box 15000, Halifax, NS B3H 4R2 Canada

**Keywords:** Anoikis, Apoptosis, ErbB2, Irf6, Breast cancer

## Abstract

**Background:**

The ability of solid tumor cells to resist anoikis, apoptosis triggered by cell detachment from the extracellular matrix (ECM), is thought to be critical for 3D tumor growth. ErbB2/Her2 oncoprotein is often overproduced by breast tumor cells and blocks their anoikis by partially understood mechanisms. In our effort to understand them better, we observed that detachment of nonmalignant human breast epithelial cells from the ECM upregulates the transcription factor Irf6. Irf6 is thought to play an important role in mammary gland homeostasis and causes apoptosis by unknown mechanisms. We noticed that ErbB2, when overproduced by detached breast epithelial cells, downregulates Irf6.

**Methods:**

To test whether ErbB2 downregulates Irf6 in human ErbB2-positive breast cancer cells, we examined the effect of ErbB2 inhibitors, such as the anti-ErbB2 antibody trastuzumab or the ErbB2/epidermal growth factor receptor small-molecule inhibitor lapatinib, on Irf6 in these cells. Moreover, we performed Irf6 IHC analysis of tumor samples derived from the locally advanced ErbB2-positive breast cancers before and after neoadjuvant trastuzumab-based therapies. To examine the role of Irf6 in anoikis of nonmalignant and ErbB2-overproducing breast epithelial cells, we studied anoikis after knocking down Irf6 in the former cells by RNA interference and after overproducing Irf6 in the latter cells. To examine the mechanisms by which cell detachment and ErbB2 control Irf6 expression in breast epithelial cells, we tested the effects of genetic and pharmacological inhibitors of the known ErbB2-dependent signaling pathways on Irf6 in these cells.

**Results:**

We observed that trastuzumab and lapatinib upregulate Irf6 in ErbB2-positive human breast tumor cells and that neoadjuvant trastuzumab-based therapies tend to upregulate Irf6 in human breast tumors. We found that detachment-induced Irf6 upregulation in nonmalignant breast epithelial cells requires the presence of the transcription factor ∆Np63α and that Irf6 mediates their anoikis. We showed that ErbB2 blocks Irf6 upregulation in ErbB2-overproducing cells by activating the mitogen-activated protein kinases that inhibit ∆Np63α-dependent signals required for Irf6 upregulation. Finally, we demonstrated that ErbB2-driven Irf6 downregulation in ErbB2-overproducing breast epithelial cells blocks their anoikis and promotes their anchorage-independent growth.

**Conclusions:**

We have demonstrated that ErbB2 blocks anoikis of breast epithelial cells by downregulating Irf6.

**Electronic supplementary material:**

The online version of this article (10.1186/s13058-018-1080-1) contains supplementary material, which is available to authorized users.

## Background

Fifteen to twenty percent of breast tumors overproduce ErbB2/Her2 receptor tyrosine kinase, which drives these cancers [1]. Overproduction of ErbB2 by the tumor cells is associated with a higher rate of disease recurrence and shorter overall survival than observed in other breast cancer types [2]. Trastuzumab, an anti-ErbB2 antibody, and lapatinib, a small-molecule inhibitor of ErbB2/epidermal growth factor receptor (EGFR), are used for treatment of ErbB2-positive cancers [3, 4]. However, many breast cancers are not cured by these drugs. For example, the malignancy recurs within 10 years in approximately 25% of women receiving trastuzumab [5]. Most of these patients die of the disease [5]. Which patients with Erbb2-positive cancers will benefit from ErbB2-trageted therapies cannot currently be predicted. Efficacious therapies of breast cancers resistant to ErbB2 antagonists are not available.

One critical feature of primary and disseminated breast tumors is their ability to grow in a 3D manner [6]. Such growth requires the ability of cancer cells to survive without adhesion to the extracellular matrix (ECM) [6]. This notion is based on the fact that normal basal and luminal breast epithelial cells are attached to the ECM in the breast, and detachment kills them by apoptosis, a phenomenon called *anoikis* [7]. In contrast, breast tumors grow, invade adjacent tissues, and metastasize as 3D cellular masses in which the cells are not properly attached to the ECM but remain viable [8]. Numerous data indicate that tumor cell anoikis resistance is critical for tumor progression. For example, the ability of cancer cells to survive and grow without adhesion to the ECM as colonies in soft agar represents one of the most stringent criteria for malignant transformation [9]. In addition, major oncoproteins such as Ras and ErbB2 block tumor cell anoikis [10, 11]. Moreover, approaches causing anoikis of tumor cells suppress their ability to form primary tumors and metastases [12]. Because ErbB2 overexpression renders breast tumor cells anoikis-resistant, mechanisms of this resistance are potential novel targets for treatment of ErbB2-positive breast cancers, and mediators of this resistance are potential biomarkers of breast tumor sensitivity to ErbB2 antagonists.

ErbB2 is a receptor tyrosine kinase that belongs to the ErbB receptor family. ErbB1/EGFR, ErbB3, and ErbB4 are other family members [13]. ErbB2 does not have a ligand and efficiently heterodimerizes with other family members once they are activated by their ligands [13]. Activated ErbB2 triggers diverse oncogenic signals, including activation of mitogen-activated protein kinases (MAPKs) [13].

ErbB2 blocks tumor cell anoikis by triggering a complex and poorly understood network of the antiapoptotic signals. We have reported that ErbB2 inhibits anoikis of breast cancer cells by downregulating the proapoptotic protein Perp [11]. Others observed that ErbB2 blocks such anoikis by downregulating the proapoptotic proteins Bim and Bmf [14]. Whether all elements of the indicated network have been discovered is unknown.

We have now identified a novel mechanism of ErbB2-dependent inhibition of breast cancer cell anoikis. This mechanism is mediated by ErbB2-induced downregulation of the transcription factor Irf6, which is thought to play an important role in the normal mammary gland homeostasis [15].

## Methods

### Materials

The following compounds were used: lapatinib (Selleckchem, Houston, TX, USA), SCH772984 (Selleckchem), and trastuzumab (Roche, Mississauga, ON, Canada) (See Additional file 1: Supplemental Methods for addtional information).

### Expression vectors

The pEGFP-C1 plasmid encoding the wild-type green fluorescent protein (GFP) was obtained from Clontech (Mountain View, CA, USA). The pBABE-hygro expression vector was purchased from Addgene (Cambridge, MA, USA). The expression vector pcDNA-HA encoding the full-length human Irf6 cDNA (pcDNA-HAIrf6) was provided by Dr. Antonio Costanzo (University of Rome, Italy). The pcDNA expression vector encoding the full-length human ΔNp63α-FLAG was obtained from Addgene. Generation of Irf6- and ΔNp63α-encoding pBabe-hygro expression vectors is described in Additional file 1: Supplemental Methods. pHIT and pVSVG retroviral vectors were provided by Dr. P. Lee (Dalhousie University, Halifax, NS, Canada). pBABE-hygro retroviral expression vector was purchased from Addgene.

### Cell culture

MCF10A cells and their derivatives MCF-ErbB2mut and MCF-ErbB2 were provided by Dr. M. Reginato (Drexel University, Philadelphia, PA, USA). The generation and use of these variants is described elsewhere [16, 17]. MCF10A cells were authenticated by the American Type Culture Collection (Manassas, VA, USA) by 17 short tandem repeat analysis. Lack of mycoplasma contamination in MCF10A, MCF-ErbB2mut, and MCF-ErbB2 cells was established by use of MycoFluor Mycoplasma Detection Kit (Molecular Probes, Eugene, OR, USA) according to the manufacturer’s instructions. MCF-ErbB2mut cells were referred to as pBabe-NeuT, MCF-ErbB2 cells as pBabe-NeuN, and MCF-MekDD as pBabe-MEK2-DD as reported previously [17]. BT-474 cells (American Type Culture Collection) were cultured in Hybri-Care medium (American Type Culture Collection), 10% FBS, 100 U/ml penicillin (Thermo Fisher Scientific, Waltham, MA, USA), 100 μg/ml streptomycin (Thermo Fisher Scientific), 0.29 mg/ml L-glutamine (Thermo Fisher Scientific). AU-565 cells (American Type Culture Collection) and HCC1419 cells (American Type Culture Collection) were cultured in RPMI 1640 medium (Thermo Fisher Scientific), 10% FBS (Sigma-Aldrich, St. Louis, MO, USA), 100 U/ml penicillin (Thermo Fisher Scientific), 100 μg/ml streptomycin (Thermo Fisher Scientific), and 0.29 mg/ml L-glutamine (Thermo Fisher Scientific). 293T cells (provided by Dr. A. Stadnyk, Dalhousie University) were cultured in DMEM (Thermo Fisher Scientific), 10% FBS, 100 U/ml penicillin, 100 μg/ml streptomycin, and 0.29 mg/ml L-glutamine. Primary human mammary epithelial cells (HMEC) (Lonza, Walkersville, MD, USA) were cultured in mammary epithelial growth medium (Lonza) supplemented with bovine pituitary extract, human epidermal growth factor, insulin, hydrocortisone, gentamicin (30 mg/ml), and amphotericin (15 mg/ml). To detach the cells from the ECM, they were plated in suspension culture above a layer of 1% sea plaque agarose polymerized in their respective culture medium not containing any additional ingredients.

### Generation of trastuzumab-resistant BT-474 cells

BT-474 cells (1 × 10^6^) were cultured in suspension for 2 weeks in the presence of 5 μg/ml trastuzumab. The surviving cells were then maintained in the monolayer culture in the presence of 5 μg/ml trastuzumab for 4 months.

### Western blot analysis

This assay was performed as described previously [10]. The following antibodies were used in this study: Anti-Irf6, anti-Blnk, anti-ErbB2, anti-RSK, and anti-phospho-RSK (all from Cell Signaling Technology, Danvers, MA, USA), anti-ΔNp63 and anti-TAp63 (BioLegend, San Diego, CA, USA), anti-CDK4 (Santa Cruz Biotechnology, Dallas, TX, USA), and anti-β-actin (Santa Cruz Biotechnology and Sigma-Aldrich).

### RNA interference

RNA interference was performed as described previously [18]. The sequences of the sense strands of the RNAs used in this study were as follows: control RNA (siCONTROL nontargeting small interfering RNA [siRNA] 1), UGUUGUUUGAGGGGAACGGTT; Irf6 siRNA 5, GGAAACUCAUCUUGGUUCA; Irf6 siRNA 7, CGUUUGAGAUCUACUUAUG; p63 siRNA 14, CGACAGUCUUGUACAAUUU; p63 siRNA 15, GAUGAACUGUUAUACUUAC. All the RNAs were purchased from Dharmacon (Lafayette, CO, USA).

### qPCR

This procedure was performed as described previously [10]. Primers used to amplify the Irf6 messenger RNA (mRNA) were as follows: CAAAACTGAACCCCTGGAGATGGA and CCACGGTACTGAAAC TTGATGTCC. Primers used to amplify the 18S rRNA were as follows: ATAGTCAAGTTCG ACCGTCTTC and GTTGATTAAGTCCCTGCCCTT.

### Transient transfection

MCF-ErbB2 cells (5 × 10^5^) were cultured in a 60-mm dish in the presence of 4 μg of either pcDNA-HA or pcDNA-HA-IRF6, 0.8 μg of the pEGFP-C1 expression vector, and 4 μg of Lipofectamine 2000 reagent in 2.0 ml of Opti-MEM medium (Thermo Fisher Scientific). Six hours later, the medium was replaced with 4 ml of the fresh medium normally used for culturing these cells. The cells were cultured for 72 h, harvested, and used for subsequent assays.

### Transduction of cells with retroviruses

293T cells (2 × 10^6^) were incubated with 5 μg of either control pBabehygro expression vector or pBabehygro-HA-Irf6 or pBabe-ΔNp63α FLAG or pBabe TAp63α expression vector and 2.5 μg of pHIT and 2.5 μg of pVSVG expression vectors encoding retroviral proteins in the presence of 20 μl of Lipofectamine 2000 reagent in 6 ml of Opti-MEM medium. The medium was changed 4 h later to DMEM containing 10% FBS. The medium was collected 48 h later and filtered through a 0.45-μm filter unit. The viral supernatant containing either the control virus or that encoding Irf6 or ΔNp63α was added to 2.5 × 10^5^ MCF-ErbB2 cells grown on a 60-mm dish in the presence of 8 μg/ml polybrene in the presence of 450 μg/ml hygromycin (in case of ΔNp63α), 300 μg/ml hygromycin (in case of ΤΑp63α) or 300 μg/ml hygromycin (in case of Irf6) for 48 h. The cells were then harvested and used for the assays described herein.

### Detection of cell survival after Irf6 RNA interference

After transfection with respective RNAs, MCF10A cells were plated in monolayers either immediately or after being cultured in suspension. Cells were allowed to form colonies for 7–10 days. The colonies were then stained with crystal violet and counted.

### Analysis of apoptosis by monitoring nuclear morphology

After transfection with siRNAs or respective expression vectors in the presence of the pEGFP-C1 expression vector, cells were cultured in suspension, harvested, and washed with PBS followed by centrifugation at 1500 rpm for 5 min at room temperature. The cell pellet was resuspended and fixed in 50 μl of 4% paraformaldehyde solution for 30 min at room temperature. The cells were then washed and resuspended in 30–50 μl of 20 μg/ml Hoechst 33258 (Molecular Probes) dissolved in PBS. Tubes with cell samples were then coded to ensure that the samples were assayed blindly, and total cell population (in case of siRNA-transfected cells) or GFP-positive (green) cells (in case of the cells transfected with the expression vectors) were analyzed by use of a Zeiss fluorescence microscope (Carl Zeiss Microscopy, Toronto, ON, Canada) for the presence of Hoechst 33258-positive (blue) condensed or fragmented nuclei (characteristic features of apoptosis) using respective light filters.

### Analysis of cell death by flow cytometry

An apoptosis detection kit (Chemicon, Temecula, CA, USA) was used. After harvesting, the cells were washed with PBS. Cells were further resuspended in the binding buffer provided by the manufacturer at a concentration of 10^6^ cells/ml. We then mixed 200 μl of the cell suspension with 3 μl of fluorescein isothiocyanate-conjugated annexin V and 2 μl of propidium iodide (PI). This solution was subsequently incubated for 15 min at room temperature. The FACSCalibur system (BD Biosciences, Franklin Lakes, NJ, USA) was used for detection of fluorescent cells.

### Soft agar colony formation assay

Cells were suspended in 2 ml of their respective growth medium containing 0.3% of melted Bacto agar. Cell suspensions were added to a 60-mm plate covered with a 2-ml layer of solidified 0.5% Bacto agar polymerized in the same medium. Cell colonies were counted after 7–10 days.

### Irf6 IHC in human breast cancer samples

Upon research ethics board approval from the local health authority, a list of patients with ErbB2-positive primary invasive breast cancer who underwent neoadjuvant chemotherapy and ErbB2-targeted therapy was obtained from the institution’s pharmacy information system (BDM Pharmacy; BDM IT Solutions, Saskatoon, SK, Canada) and a laboratory information system (Cerner Millennium; Cerner, North Kansas City, MO, USA). All patients were treated with neoadjuvant trastuzumab plus six cycles of fluorouracil, epirubicin, cyclophosphamide, and docetaxel prior to surgical resection of the tumor. A cohort of 11 consecutive cases was randomly selected from the 2011–2015 list for assessment of Irf6 expression in order to ensure an adequate follow-up interval. Representative tissue blocks from the diagnostic core biopsies and postneoadjuvant excisional specimens were selected in each case for Irf6 IHC after review of the H&E- as well as anti-ErbB2-stained IHC slides to confirm diagnosis and ErbB2 positivity. Formalin-fixed, paraffin-embedded core biopsies and excisional specimens underwent heat-induced epitope retrieval for 24 min in Cell Conditioning 1 (Ventana Medical Systems, Tucson, AZ, USA) followed by 32-min incubation in 1:100 dilution of primary Irf6 antibody (clone 2A12; MyBioSource, San Diego, CA, USA). The reaction was detected using the OptiView polymer detection system on a Ventana Benchmark Ultra platform (Ventana Medical Systems). Cells were also counterstained with hematoxylin. Nuclear positivity was scored on the preneoadjuvant core biopsies and postneoadjuvant excisional specimens following Irf6 IHC staining by manually counting positively stained nuclei as a percentage of all breast cancer cells. At least 400 cells were counted in each case, with the exception of one sample in which only 53 malignant cells were counted after neoadjuvant therapy (all cells were counted).

### Statistical analysis

Statistical analysis of the data in Fig. 7a was performed by using the unpaired Student’s *t* test. Statistical analysis of all other data was performed by using the chi-square test for goodness of fit.

## Results

### ErbB2 downregulates Irf6 in breast epithelial cells

**Fig. 1:**
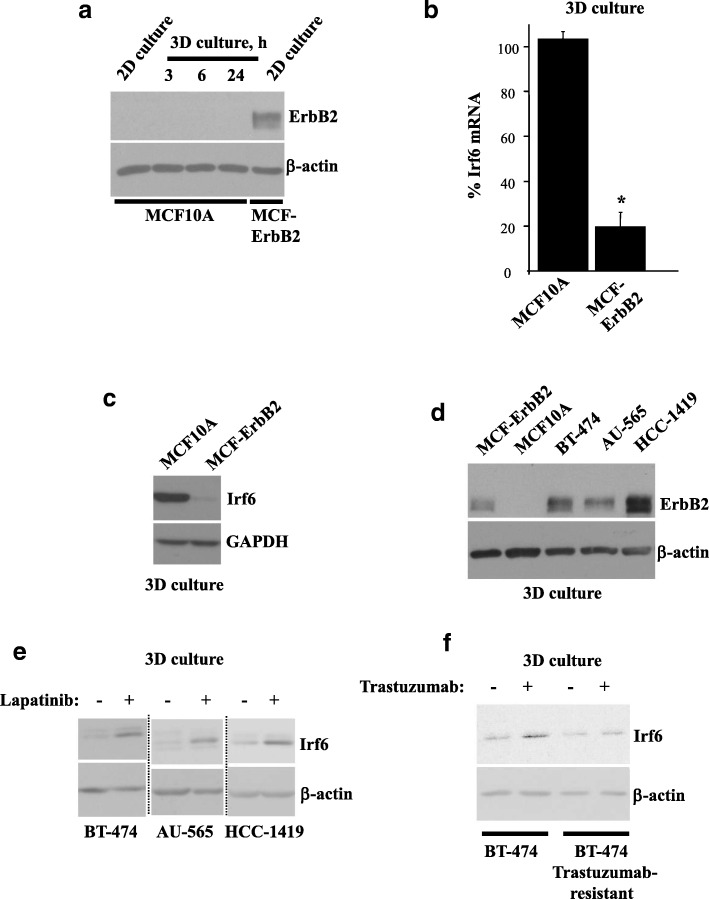
ErbB2 downregulates Irf6 in detached breast epithelial cells. **a** MCF10A and MCF-ErbB2 cells were cultured attached to (2D culture) or detached from (3D culture) the extracellular matrix for the indicated times and assayed for ErbB2 levels by Western blotting. **b** MCF10A and MCF-ErbB2 cells were cultured in 3D culture for 2 h, and Irf6 messenger RNA (mRNA) levels were analyzed in the cells by qPCR. Irf6 mRNA levels were normalized to those of 18S ribosomal RNA (determined by qPCR). The resulting Irf6 mRNA levels in one of the replicates derived from MCF10A cells were designated as 100%. Results represent the average of two independent experiments plus the SD. * *p* < 0.05. **c** MCF10A and MCF-ErbB2 cells were kept as in (**b**) for 3 h and assayed for Irf6 levels by Western blotting. Glyceraldehyde 3-phosphate dehydrogenase (GAPDH) served as a loading control. **d** MCF10A and MCF-ErbB2, as well as human breast carcinoma cells BT-474, AU-565, and HCC-1419, were kept in 3D culture for 2 h and assayed for ErbB2 levels by Western blotting. **e** Indicated cells were cultured in 3D culture for 48 h in the absence or presence of 1 μM lapatinib and assayed for Irf6 expression by Western blotting. **f** BT-474 cells or their trastuzumab-resistant variant were kept in 3D culture for 48 h in the absence or presence of 5 μg/ml trastuzumab and assayed for Irf6 expression by Western blotting. β-actin was used as a loading control in **a** and **d–f**, and GAPDH served as a loading control in **c**

In an effort to identify the mechanisms by which ErbB2 blocks breast cancer cell anoikis, we used MCF10A cells, which are spontaneously immortalized highly anoikis-susceptible human nonmalignant breast epithelial cells [11]. They do not produce ErbB2 in 2D culture (when the cells are attached to the ECM) or in 3D culture (when they are detached from the ECM) (Fig. 1a, d). We also used a published anoikis-resistant derivative of MCF10A cells, MCF-ErbB2 cells, which were generated by infection of MCF10A cells with a retrovirus encoding the wild-type ErbB2 [16, 17] (Fig. 1a, d). We noticed that ErbB2 strongly downregulates Irf6 mRNA and protein in MCF10A cells in 3D culture (Fig. 1b, c).

Irf6 is a member of the interferon-regulatory factor family of transcription factors [19]. Irf6 upregulation in cells can kill them by apoptosis [19, 20]. Importantly, Irf6 is upregulated in the breast during mammary gland regression upon weaning, and such regression likely involves breast epithelial cell anoikis [21]. Moreover, Irf6 protein tends to be downregulated in breast, nasopharyngeal, and squamous cell carcinomas [22–24]. Irf6 can be phosphorylated at several amino acid residues and is often detected as a doublet on Western blots [25]. At least two of these phosphorylation events are required for Irf6 transcriptional activity [26].

We observed that lapatinib, a small-molecule ErbB2/EGFR inhibitor used for treatment of ErbB2-positive breast cancer, strongly upregulates Irf6 in detached ErbB2-positive human breast cancer cells BT-474, AU-565, and HCC-1419 (Fig. 1d, e) [4]. Furthermore, we found that the anti-ErbB2 antibody trastuzumab used for ErbB2-positive breast cancer treatment upregulates Irf6 in BT-474 cells much more noticeably than in their variant selected for trastuzumab resistance by prolonged cell exposure to trastuzumab in 3D culture (Fig. 1f) [3]. Thus, ErbB2 downregulates Irf6 in detached human breast cancer cells.

### Detachment-induced Irf6 upregulation contributes to anoikis of nonmalignant breast epithelial cells

**Fig. 2:**
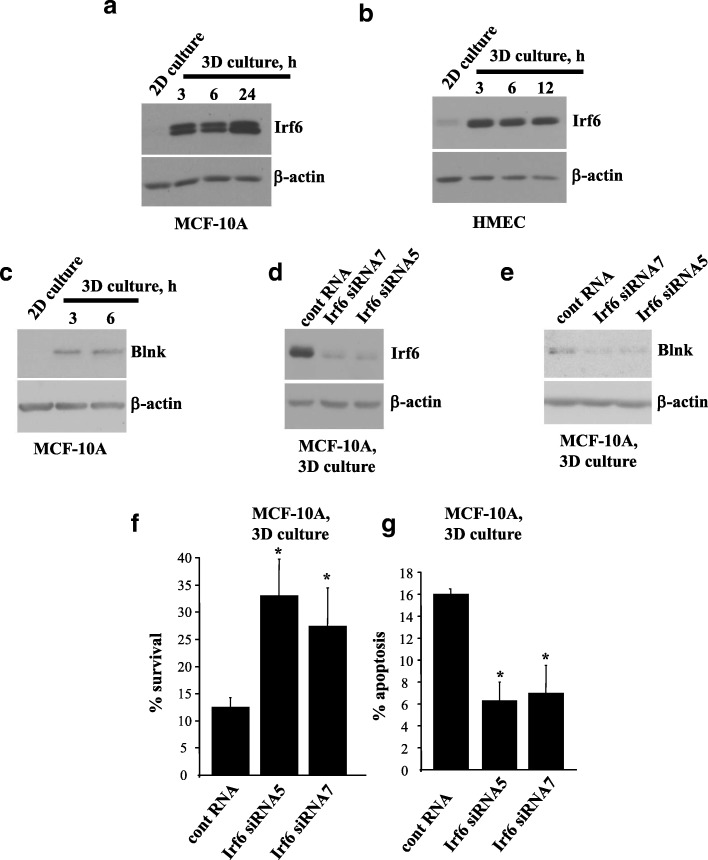
Detachment-induced upregulation of Irf6 is required for anoikis of nonmalignant breast epithelial cells. **a**, **b** MCF10A cells (**a**) or human mammary epithelial cells (HMEC) (**b**) were kept attached to (2D culture) or detached from (3D culture) the extracellular matrix for the indicated times and assayed for Irf6 levels by Western blotting. **c** MCF10A cells were kept in 2D or 3D culture for the indicated times and assayed for Blnk levels by Western blotting. **d**, **e** MCF10A cells transfected with 100 nM control RNA or Irf6-specific small interfering RNA 5 or 7 were detached for 3 h and assayed for Irf6 (**d**) or Blnk (**e**) expression by Western blotting. β-actin was used as a loading control in **a–e**. **f** MCF10A cells treated as in (**d**) were allowed to form colonies in monolayers immediately or after 72 h of detachment. % survival is the percentage of colony number formed by the cells plated in monolayers immediately after transfection. The data represent the average of three independent experiments plus the SE. **g** MCF10A cells treated as in (**c**) were detached for 48 h, then cell nuclei were stained with Hoechst 33258, and the percentage of cells with condensed and/or fragmented nuclei (characteristic features of apoptosis) in the total cell population (% apoptosis) was determined. The data represent the average of two independent experiments plus the SD. * *p* < 0.05

We further found that detachment of MCF10A cells or that of anoikis-susceptible nonmalignant primary HMEC from the ECM upregulates Irf6 (Fig. 2a, b) [27]. Thus, detachment-induced Irf6 upregulation is not unique to MCF10A cells.

Others found that Irf6 knockdown in human keratinocytes by siRNAs downregulates several mRNAs, including that encoding the proapoptotic protein Blnk [24, 28]. We noticed that, similar to what was observed in the case of Irf6, detachment from the ECM strongly upregulates Blnk in MCF10A cells (Fig. 2c). In addition, we found that Irf6 knockdown in MCF10A cells by two different siRNAs (Fig. 2d) downregulates Blnk in 3D culture (Fig. 2e), indicating that Irf6 is capable of controlling gene expression under these conditions. Moreover, we observed that Irf6 knockdown increases survival of detached MCF10A cells (Fig. 2f) and substantially reduces their apoptosis in 3D culture (Fig. 2g). Hence, detachment-induced Irf6 upregulation contributes to anoikis of nonmalignant breast epithelial cells.

### ErbB2-induced Irf6 downregulation is required for anoikis resistance of breast epithelial cells

**Fig. 3:**
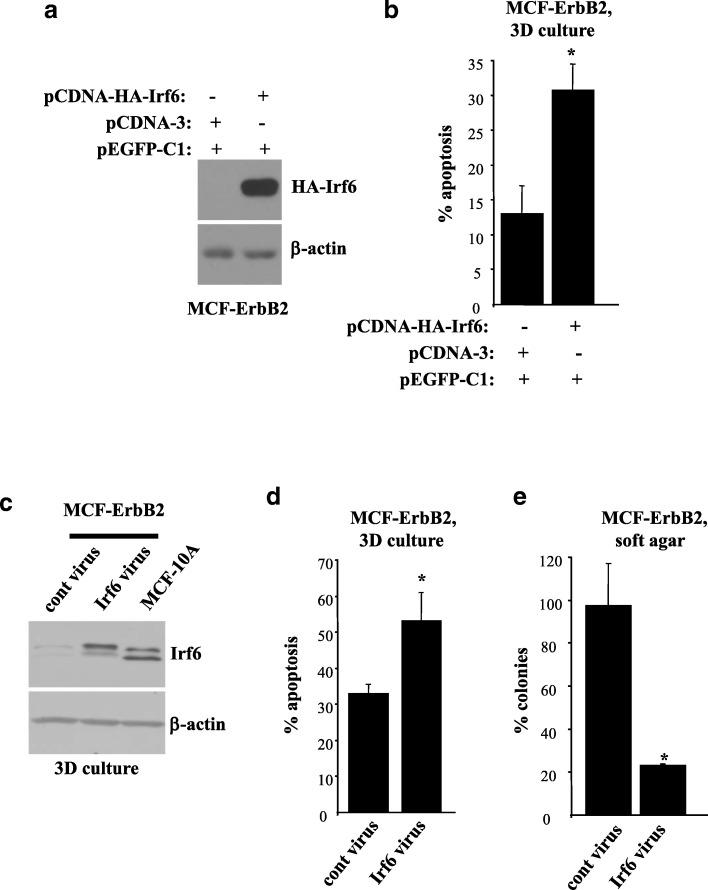
Downregulation of Irf6 is required for ErbB2-induced anoikis resistance of breast epithelial cells. **a** MCF-ErbB2 cells were transiently transfected or not with a control vector (pcDNA3) or pcDNA3 vector encoding hemagglutinin (HA)-tagged Irf6 (pcDNA-HA-Irf6) and a vector encoding green fluorescent protein (GFP; vector pEGFP-C1) and assayed 24 h later for HA-Irf6 expression by Western blotting using an anti-HA antibody. **b** Cells treated as in (**a**) were cultured detached from the extracellular matrix (in 3D culture) for 72 h, cell nuclei were stained with Hoechst 33258, and the percentage of GFP-positive cells with condensed and/or fragmented nuclei (characteristic features of apoptosis) was determined as the percentage of the cells with such nuclei in a population of GFP-positive cells. **c** MCF-ErbB2 cells infected with the control or the Irf6-encoding Moloney murine leukemia virus were kept in 3D culture for 3 h along with MCF10A cells and assayed for Irf6 levels by Western blotting. **d** MCF-ErbB2 cells treated as in (**c**) were kept in 3D culture for 24 h, stained with propidium iodide (PI), and assayed for annexin V binding by flow cytometry. % Apoptosis is the sum of the percentage of annexin V-positive/PI-negative cells and that of annexin V-positive/PI-positive cells. **e** MCF-ErbB2 cells treated as in (**c**) were allowed to form colonies in soft agar. The number of colonies formed by one of the replicates of the control cells was designated as 100%. The data in (**b**, **d**) are the average of two independent experiments plus the SD, and those in (**e**) are the average of three independent experiments plus the SE. β-actin was used as a loading control in (**a**, **c**). * *p* < 0.05

To reverse the effect of ErbB2 on Irf6, we transiently expressed ectopic hemagglutinin (HA)-tagged Irf6 in MCF-ErbB2 cells together with a GFP to visualize the transfected cells by fluorescence microscopy (Fig. 3a). Exogenous Irf6 noticeably increased apoptosis of MCF-ErbB2 cells in 3D culture (Fig. 3b).

To examine the role of Irf6 in the regulation of anoikis of ErbB2-producing cells by a complementary technique, we infected MCF-ErbB2 cells with a retrovirus encoding HA-tagged Irf6 (Fig. 3c). Exogenous Irf6 significantly increased apoptosis of MCF-ErbB2 cells in 3D culture (Fig. 3d) and noticeably reduced their clonogenicity without adhesion to the ECM in soft agar (the ability to grow in agar is a well-known consequence of cancer cell anoikis resistance [11]) (Fig. 3e). Hence, ErbB2-induced Irf6 downregulation is required for anoikis resistance of ErbB2-overproducing breast epithelial cells.

### Detachment-induced Irf6 upregulation in nonmalignant breast epithelial cells requires the presence of ∆Np63α

One protein that promotes Irf6 transcription by binding the Irf6 promoter is ∆Np63, a member of the p53 family of transcription factors [29]. The p63 gene is transcribed from two different promoters to yield the transcription factors TAp63 or ∆Np63 containing DNA-binding and oligomerization domains [29]. In addition, TAp63 has an N-terminal transactivation domain. Both TAp63 and ∆Np63 exist as α-, β-, or γ-isoforms generated via alternative splicing [29]. Importantly, ErbB2 causes loss of all p63 isoforms in a mouse model of breast cancer, and none of the p63 isoforms are expressed in human breast tumors [30]. ∆Np63 can trigger pro- and antiapoptotic signals [31, 32]. Enforced downregulation of ∆Np63α in MCF10A cells causes their epithelial-to-mesenchymal transition, whereas ErbB2- or Ras oncoprotein-induced ∆Np63α downregulation in MCF10A and other epithelial cell lines increases cell migration and metastatic potential [33, 34].

**Fig. 4:**
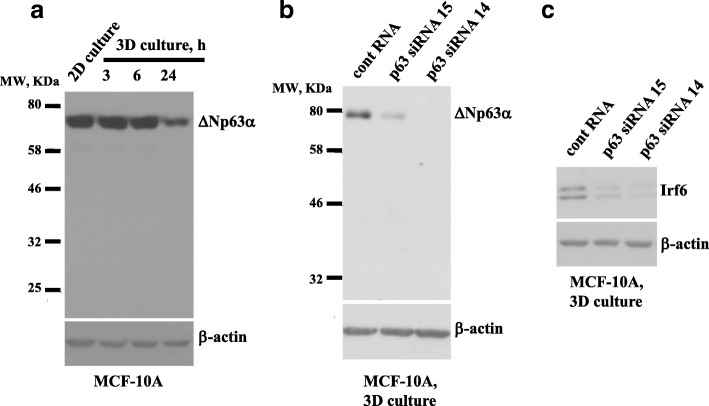
Detachment-induced upregulation of Irf6 in nonmalignant breast epithelial cells requires the presence of ΔNp63α. **a** MCF10A cells were cultured attached to (2D culture) or detached from (3D culture) the extracellular matrix for the indicated times and assayed for ΔNp63 levels by Western blotting. **b**, **c** MCF10A cells transfected with 100 nM control RNA (cRNA) or p63-specific small interfering RNA (p63siRNA) 14 or 15 were kept in 3D culture for 3 h and assayed for ΔNp63 (**b**) or Irf6 (**c**) expression by western blot. β-actin was used as a loading control

We investigated the status of ∆Np63 in MCF10A cells by using ∆Np63-specific antibody validated for the detection of the ∆Np63 isoforms [35]. Similar to published observations, we found that attached MCF10A cells produce only ∆Np63α (Fig. 4a) [34]. We further noticed that when MCF10A cells detach from the ECM, ∆Np63α levels remain unchanged for at least 6 h but decline at 24 h of 3D culture (Fig. 4a). The antibody validated by others for the detection of TAp63 did not detect any TAp63 species in MCF10A cells in 2D or 3D culture (Additional file 2: Figure S1) [35]. Because Irf6 is upregulated in these cells as early as 3 h of 3D culture (Fig. 2a), we reasoned that ∆Np63α could mediate detachment-induced Irf6 upregulation, while ∆Np63a levels in the cells in 3D culture are still high. Indeed, knockdown of p63 by two different siRNAs (Fig. 4b) caused noticeable Irf6 downregulation in MCF10A cells in 3D culture (Fig. 4c and Additional file 3: Figure S2). Importantly, although ∆Np63α levels are relatively elevated in the MCF10A cells in 2D culture (Fig. 4a), this is insufficient for Irf6 upregulation (Fig. 2a). Therefore, Irf6 is likely upregulated in these cells by yet unidentified detachment-induced signals that can upregulate Irf6 only in the presence of ∆Np63α.

### ErbB2-induced downregulation of Irf6 in breast epithelial cells is mitogen-activated protein kinase-dependent

**Fig. 5:**
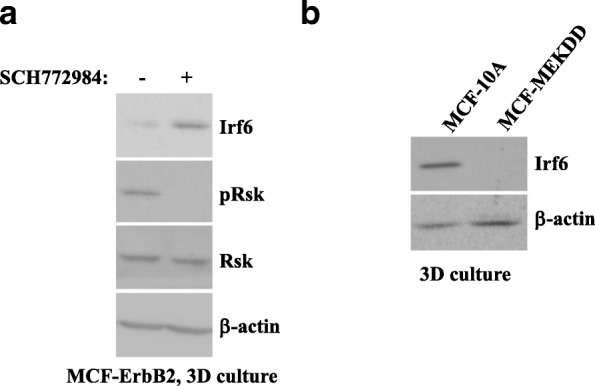
ErbB2-induced Irf6 downregulation occurs in a mitogen-activated protein kinase-dependent manner. **a** MCF-ErbB2 cells were cultured for 24 h detached from the extracellular matrix (3D culture) in the presence of dimethyl sulfoxide or 1 μM SCH772984 and assayed for Irf6 expression by Western blotting. The membrane was reprobed with an anti-phospho-Rsk (pRsk) antibody and then an anti-Rsk antibody. **b** MCF10A cells and a variant of MCF10A cells obtained by infection of these cells with a retrovirus carrying constitutively active Mek2 mutant (MCF-MekDD) were kept in 3D culture for 3 h and assayed for Irf6 expression by Western blotting. β-actin was used as a loading control in (**a**) and (**b**)

The MAPKs Erk1 and Erk2 are major mediators of ErbB2 signaling [36]. ErbB2 induces MAPK signaling by activating a GTPase Ras, which then activates the protein kinase Raf [36]. Raf further phosphorylates and thereby activates the protein kinases Mek1 and Mek2. This allows the Mek kinases to phosphorylate and activate Erk1 and Erk2 [36]. Erk1 and Erk2 then phosphorylate and change the activity of various proteins [36]. We found that treatment with an Erk inhibitor, SCH772984, significantly reduced phosphorylation of the Erk substrate Rsk and noticeably upregulated Irf6 in MCF-ErbB2 cells in 3D culture (Fig. 5a) [37, 38].

We further found that a published derivative of MCF10A cells, MCF-MekDD, which we generated by infection of MCF10A cells with a retrovirus encoding an activated mutant of Mek (an Erk activator), displays significantly lower Irf6 levels than the parental MCF10A cells in 3D culture (Fig. 5b) [39]. Thus, Mek activation is sufficient for Irf6 downregulation in breast epithelial cells detached from the ECM. Collectively, our data indicate that ErbB2 downregulates Irf6 in breast epithelial cells in a MAPK-dependent manner.

### ErbB2 inhibits signals that promote ∆Np63α-dependent Irf6 upregulation in breast epithelial cells detached from the ECM

**Fig. 6:**
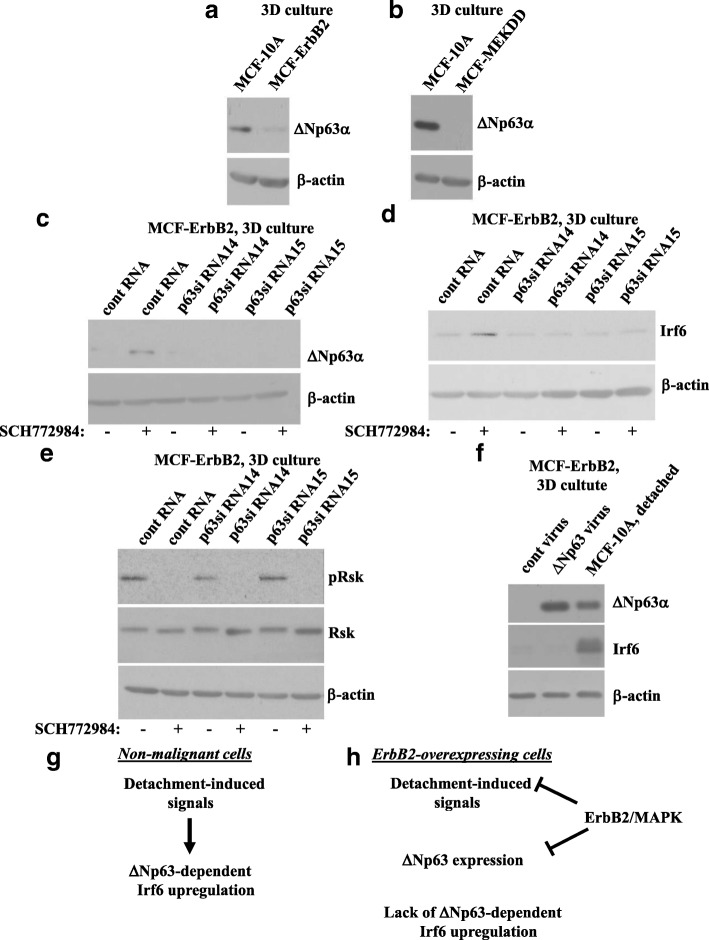
Erk blocks ∆Np63α-dependent signals that upregulate Irf6 in detached ErbB2-overproducing breast epithelial cells. **a**, **b** MCF10A and MCF-ErbB2 cells (**a**) or MCF10A and MCF-MekDD cells (**b**) were cultured detached from the extracellular matrix (3D culture) for 3 h and assayed for ΔNp63 levels by Western blotting. **c**–**e** MCF-ErbB2 cells transfected with 100 nM control RNA (cRNA) or p63-specific small interfering RNA (p63siRNA) 14 or 15 were kept in 3D culture for 24 h in the presence of dimethyl sulfoxide or 1 μM SCH772984 and assayed for ΔNp63 (**c**), Irf6 (**d**), or phospho-Rsk (pRsk) and Rsk (**e**) expression by Western blotting. **f** MCF-ErbB2 cells were infected with the control or the ΔNp63α-encoding Moloney murine leukemia virus, kept in 3D culture for 3 h along with MCF10A cells, and assayed for ΔNp63 levels by Western blotting. β-actin was used as a loading control in (**a–f**). **g**, **h** Schematic representation of events that take place in detached nonmalignant (**g**) and ErbB2-overproducing (**h**) breast epithelial cells. **g** Detachment-induced signals can upregulate Irf6 in the nonmalignant cells only in the presence of ΔNp63α. **h** ErbB2 blocks both the indicated detachment-induced signals and ΔNp63α expression in detached breast cancer cells. In the absence of the indicated detachment-induced signals, Irf6 is not upregulated in ErbB2-overproducing cells

Because ∆Np63α is required for detachment-induced Irf6 upregulation in nonmalignant breast epithelial cells (Fig. 4), we investigated the role of ∆Np63α in the effect of ErbB2 on Irf6. We noticed that both ErbB2 and an activated Mek mutant (Fig. 6a, b and Additional file 4: Figure S3) downregulate ∆Np63α in MCF10A cells in 3D culture. We further observed that the Erk inhibitor SCH772984 upregulates ∆Np63α in MCF10-ErbB2 cells in 3D culture (Fig. 6c). Moreover, when this upregulation was blocked by two different p63-specific siRNAs (Fig. 6c), the Erk inhibitor failed to upregulate Irf6 in the cells (Fig. 6d). As expected, the inhibitor blocked phosphorylation of the Erk substrate Rsk in all cases (Fig. 6e). Thus, ErbB2/MAPK downregulates Irf6 by suppressing ∆Np63α-dependent signals in cells detached from the ECM. It is noteworthy that in the case of nonmalignant breast epithelial cells, detachment-induced signals can upregulate Irf6 only in the presence of ∆Np63α (Figs. 4 and 6d). Hence, ErbB2/MAPK signaling could downregulate Irf6 in MCF-ErbB2 cells in 3D culture either by blocking the indicated signals or by downregulating ∆Np63α itself. To distinguish between these possibilities, we infected MCF-ErbB2 cells with a retrovirus encoding ∆Np63α (Fig. 6f). We found that ectopic ∆Np63α does not upregulate Irf6 in these cells in 3D culture (Fig. 6f). Thus, ErbB2/MAPK-driven signals block detachment-induced events that promote ∆Np63α-dependent Irf6 upregulation and, in addition, downregulate ∆Np63α itself in MCF-ErbB2 cells detached from the ECM (*see* Fig. 6g, h for models describing these scenarios). When Erk activity is blocked, both of these events are reversed, and Irf6 is upregulated (Fig. 6c, d). However, in the absence of the indicated detachment-induced signals, ectopic ∆Np63α by itself cannot upregulate Irf6.

### Neoadjuvant ErbB2-targeted therapies are accompanied by Irf6 upregulation in patient-derived breast tumors

One approach to testing whether ErbB2 downregulates Irf6 in human breast cancer is to examine whether therapies based on the use of a therapeutic anti-ErbB2 antibody trastuzumab upregulate Irf6 in patients’ tumors. Addressing this question requires access to tumor samples before and after the treatment. These samples can be derived from patients with ErbB2-positive, locally advanced breast cancer. Such patients normally receive neoadjuvant trastuzumab and chemotherapy for approximately 3 months, followed by tumor resection and further trastuzumab treatment for up to 9 months [40].

**Fig. 7:**
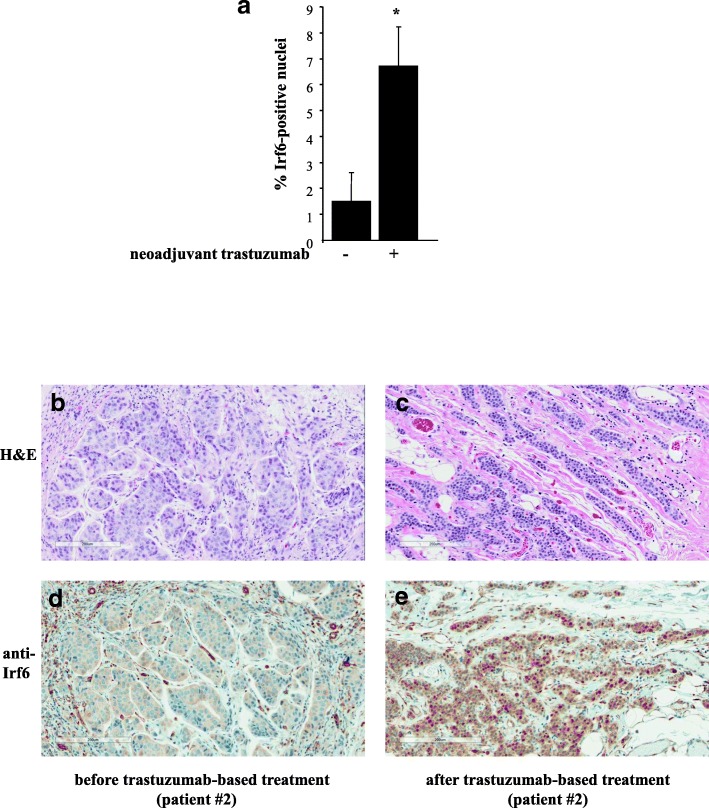
Irf6 is upregulated in breast tumor cells after neoadjuvant trastuzumab-based therapy. Formalin-fixed, paraffin-embedded tumor sections obtained from patients before and after the therapy were stained with an anti-Irf6 antibody. **a** Percentage of tumor cells with Irf6-positive nuclei before the treatment with neoadjuvant trastuzumab and after the treatment is shown. The data represent the average (plus the SE) of respective percentages observed in nine patients. **b–e** Representative samples obtained from patient 2 before (**b**, **d**) and after (**c**, **e**) the treatment are shown. The samples were stained with hematoxylin (blue) and eosin (red) (H&E) (**b**, **c**) or with an anti-Irf6 antibody (brown) and counterstained with hematoxylin (blue) (**d**, **e**)

To begin to test the effect of trastuzumab-based therapies on Irf6 in human breast tumors, we used a pilot cohort of 11 patients with locally advanced breast cancers treated at the QEII Health Centre, Halifax, NS, Canada, with neoadjuvant trastuzumab and chemotherapy prior to surgical tumor resection. We obtained tissue sections from the diagnostic core biopsies and from the postneoadjuvant therapy excisional specimens and assessed Irf6 levels in these sections by IHC. In all 11 cases, breast cancer cells displayed very low cytoplasmic Irf6 staining. Likewise, the pretreatment samples showed poorly detectable nuclear Irf6 staining. In contrast, 9 of 11 (81%) of the posttreatment samples displayed various degrees of increased nuclear Irf6 staining (Fig. 7a). Overall, the percentage of Irf6 positive nuclei was increased approximately 4.5-fold after the treatment. Representative IHC data are shown in Fig. 7b–e (additional IHC data are shown in Additional file 5: Figure S4, Additional file 6: Figure S5, Additional file 7: Figure S6). Thus, Irf6 upregulation in patient-derived breast tumors is associated with ErbB2-targeted therapies.

## Discussion

We have identified a novel mechanism of ErbB2-dependent inhibition of anoikis of breast epithelial cells involving ErbB2-dependent downregulation of the transcription factor Irf6. Our results are relevant to breast cancer because Irf6 tends to be downregulated in this malignancy [22].

Others have observed that Irf6 is upregulated in the breast during mammary gland involution upon cessation of lactation [15]. This involution is accompanied by the production of ECM-degrading proteases by the breast and is likely, at least in part, mediated by breast epithelial cell anoikis [21]. The possibility that Irf6 mediates such anoikis is supported by our data. We found that when nonmalignant breast epithelial cells detach from the ECM, Irf6 is upregulated and contributes to their apoptosis.

It has been reported that Irf6 expression is required for cell proliferation in certain contexts (e.g., downstream of the Notch receptor) but that Irf6 upregulation kills cells by apoptosis [20, 41]. Our data are consistent with the latter findings in that detachment-induced Irf6 upregulation causes anoikis of breast epithelial cells.

We have shown that detachment-induced Irf6 upregulation in nonmalignant breast epithelial cells requires the presence of the transcription factor ∆Np63α. Perhaps not by coincidence, ∆Np63 and other p63 isoforms are typically not produced by breast cancer cells [30].

We have demonstrated that ErbB2-dependent Irf6 regulation is mediated by the MAPKs. These observations are consistent with the findings made by us and others indicating that MAPKs trigger diverse antianoikis signals in breast epithelial cells [11, 16].

ErbB2-driven breast tumor cell anoikis resistance is thought to be a prerequisite for breast cancer progression [6, 8]. Of note, we found that ErbB2-targeted drugs such as trastuzumab upregulate Irf6 in trastuzumab-sensitive but not in trastuzumab-resistant ErbB2-producing detached human breast cancer cells. Moreover, we observed that neoadjuvant trastuzumab-based treatments of patients with locally advanced breast cancer tend to cause Irf6 upregulation in tumors. Thus, ErbB2 likely downregulates Irf6 levels in patients’ tumors, and Irf6 upregulation may be associated with trastuzumab sensitivity of breast cancer cells.

Cancer relapses in about 30% of patients with locally advanced breast cancer who receive neoadjuvant trastuzumab-based therapy followed by tumor surgical excision and trastuzumab treatment [40]. Whether a relapse will occur cannot currently be reliably predicted. Trastuzumab can have serious side effects (e.g., cardiotoxicity) and is costly [42]. Exploring whether Irf6 can serve as a biomarker of breast cancer trastuzumab sensitivity represents a promising direction for our future studies. If trastuzumab-driven Irf6 upregulation in the primary tumor following neoadjuvant trastuzumab-based treatments signifies increased overall patient survival, future patients whose tumors show such upregulation might be expected to benefit from the postsurgery trastuzumab treatment more than those whose tumors do not show Irf6 upregulation.

## Conclusions

We have demonstrated that anoikis of nonmalignant breast epithelial cells is mediated by detachment-induced Irf6 upregulation. We have also shown that ErbB2, a major oncoprotein, downregulates Irf6 in breast cancer cells growing in a 3D manner. We have further demonstrated that the effect of ErbB2 on Irf6 can be blocked by ErbB2-targeted drugs such as trastuzumab in cultured breast cancer cells and in patients’ tumors. Resistance to this drug is associated with lack of Irf6 upregulation. Finally, we have established that ErbB2-dependent Irf6 downregulation is required for the ability of ErbB2-overproducing breast epithelial cells to resist anoikis and grow in a 3D, anchorage-independent manner.

## Additional files

Additional file 1: Supplementary Methods. (DOC 42 kb)

Additional file 2: **Figure S1.** TAp63 is not detectable in MCF10A cells. **a** MCF10A cells were cultured attached to (2D culture) or detached from (3D culture) the ECM for the indicated times and assayed for TAp63 levels by Western blotting by use of a TAp63-specific antibody. **b** To ensure that the TAp63-specific antibody was capable of recognizing TAp63 in our experimental conditions, we infected MCF-ErbB2 cells with a control or a TAp63a-encoding retroviruses. TAp63 levels in the cells were assayed by Western blotting using the indicated antibody. β-actin was used as a loading control. (PPT 188 kb)

Additional file 3: **Figure S2.** p63-specific siRNAs downregulate Irf6 in MCF10A cells in 3D culture. MCF10A cells transfected with 100 nM control RNA (cRNA) or p63-specific siRNA (p63siRNA) 14 or 15 were kept in 3D culture for 3 h and assayed for Irf6 expression by Western blotting. β-actin was used as a loading control in one experiment, and α-tubulin was used as a loading control in another independent experiment. Films were scanned, and densitometric analysis of the resulting digital images was performed. Irf6 protein levels were normalized to those of the loading controls. The data represent the average of two independent experiments plus the SD. * *p* < 0.05. (PPT 53 kb)

Additional file 4: **Figure S3.** ErbbB2 and Mek downregulate ΔNp63α in detached breast epithelial cells. MCF10A and MCF-ErbB2 cells (**a**–**c**) or MCF10A and MCF-MekDD cells (**b**–**d**) were cultured detached from the ECM (3D culture) for the indicated times and assayed for ΔNp63 levels by Western blotting. β-actin was used as a loading control. Fragments of panels **a** and **d** showing ΔNp63 levels are displayed in Fig. 6a and b, respectively. (PPT 318 kb)

Additional file 5: **Figure S4.** Irf6 is upregulated in breast tumor cells after neoadjuvant trastuzumab-based therapy. Formalin-fixed, paraffin-embedded tumor sections obtained from patient 4 before (**a**, **c**) and after (**b**, **d**) the therapy were stained with an anti-Irf6 antibody. The samples were stained with hematoxylin (blue) and eosin (red) (H&E) (**a**, **b**) or with an anti-Irf6 antibody (brown) (**d**, **e**) and counterstained with hematoxylin (blue). (PPT 1479 kb)

Additional file 6: **Figure S5.** Irf6 is upregulated in breast tumor cells after neoadjuvant trastuzumab-based therapy. Formalin-fixed, paraffin-embedded tumor sections obtained from patient 5 before (**a**, **c**) and after (**b**, **d**) the therapy were stained with an anti-Irf6 antibody. The samples were stained with hematoxylin (blue) and eosin (red) (H&E) (**a**, **b**) or with an anti-Irf6 antibody (brown) (**d**, **e**) and counterstained with hematoxylin (blue). (PPT 1471 kb)

Additional file 7: **Figure S6.** Irf6 is upregulated in breast tumor cells after neoadjuvant trastuzumab-based therapy. Formalin-fixed, paraffin-embedded tumor sections obtained from patient 7 before (**a**, **c**) and after (**b**, **d**) the therapy were stained with an anti-Irf6 antibody. The samples were stained with hematoxylin (blue) and eosin (red) (H&E) (**a**, **b**) or with an anti-Irf6 antibody (brown) (**d**, **e**) and counterstained with hematoxylin (blue). (PPT 1498 kb)

## Abbreviations

ECM: Extracellular matrix
EGFR: Epidermal growth factor receptor
GAPDH: Glyceraldehyde 3-phosphate dehydrogenase
GFP: Green fluorescent protein
HA: Hemagglutinin
HMEC: Human mammary epithelial cells
MAPK: Mitogen-activated protein kinase
mRNA: Messenger RNA
PI: Propidium iodide
siRNA: Small interfering RNA
