# Supplementary material for: ErbB2-driven downregulation of the transcription factor Irf6 in breast epithelial cells is required for their 3D growth
Source: Breast Cancer Res. 2018 Dec 13;20:151. doi: 10.1186/s13058-018-1080-1 (PMC6293553; doi:10.1186/s13058-018-1080-1)
Supplement: Supplementary file 1 — Supplementary Methods. (DOC 42 kb) [file 13058_2018_1080_MOESM1_ESM.doc]

**Supplementary Methods**

**Generation of Irf6- and ΔNp63α-encoding pBabe-hygro expression vectors**

To insert the HA-Irf6 cDNA fragment into the pBABE-hygro expression vector the HA-Irf6 cDNA carrying SalI restriction sites at both ends was generated by PCR using pcDNA-HAIrf6 expression vector described above as a template and the oligonucleotides TTAGTCGACATGGCTTACCCA as a forward primer, and TTAGTCGACTTACTGGGGAGG as a reverse primer. The resulting PCR product was purified in 0.8% agarose gel using QIAquick Gel Extraction Kit (Qiagen, Toronto, ON, Canada). The amplified HA-Irf6 cDNA fragment was ligated into the pCR2.1-TOPO vector using TOPO TA Cloning kit (Invitrogen, Carlsbad, CA), and the insert was sequenced using the M13 forward primer GTAAAACGACGGCCAG and the M13 reverse primer CAGGAAACAGCTATGAC at Clinical Genomic Centre at the Mountain Sinai Hospital (Toronto, ON, Canada). The vector was then digested with SalI restriction enzyme (New England Biolabs, Whitby, ON, Canada), the resulting Irf6 cDNA was purified in 0.8% agarose gel. The SalI-digested pBabe-hygro expression vector was dephosphorylated by alkaline phosphatase (New England Biolabs, Whitby, ON, Canada). The indicated HA-IRF6 cDNA was ligated to the SalI-digested pBabe-hygro vector by use of T4 DNA ligase (New England Biolabs, Whitby, ON, Canada). The *E. coli* competent cells were transformed with the ligation mixture, the DNA was extracted from the resulting colonies and the presence of the HA-Irf6 cDNA was confirmed by digestion of the indicated vector with the EcoRI restriction enzyme.

To generate ΔNp63α-encoding retrovirus ΔNp63α-FLAG cDNA carrying BamHI and SalI restriction sites at the 5' and 3' ends respectively, was generated by PCR using the forward primer TTAGGATCCATGTTGTACCTG, the reverse primer TATGTCGACCTACTACTT GTCATCGTC and the pcDNA ΔNp63α-FLAG as a template. The PCR product was then purified in a 0.8% agarose gel as above. The pBabe-hygro vector and the indicated ΔNp63α-FLAG DNA fragment were digested with BamHI (New England Biolabs, Whitby, ON, Canada) and SalI (New England Biolabs, Whitby, ON, Canada) restriction enzymes, the digested fragments were purified in 0.8% agarose gel as above and ligated to each other by T4 DNA ligase. The *E.coli* competent cells were further transformed with the ligation mixture, the DNA was extracted from the resulting colonies and the presence of the ΔNp63α-FLAG in the vector was confirmed by sequencing using the sequencing primers CCGTCTCTCCCCCTTGAAC, GCAGAACAGCGTCACGGC, GCCCAGTATGTAGAAGATCCCA, and GGGCCGT GAGACTTATGAAA at the Clinical Genomics Centre at the Mountain Sinai Hospital (Toronto, ON, Canada). To generate the TAp63-encoding retrovirus the BABE-hygro and TAp63alpha-FLAG expression vectors (Addgene, Cambridge, MA, USA) were each digested with BamHI (New England Biolabs, Whitby, ON, Canada) and BsaAI (New England Biolabs, Whitby, ON, Canada) and the digested pBabe-hygro vector was treated with the alkaline phosphatase (New England Biolabs, Whitby, ON, Canada). The digested pBabe-hygro and TAp63alpha-FLAG vectors were then purified in 0.8% agarose gel using QIAquick Gel Extraction Kit (Qiagen, Toronto, ON, Canada) and ligated to each other with the T4 DNA ligase (New England Biolabs, Whitby, ON, Canada). The *E.coli* competent cells were further transformed with the ligation mixture, bacterial colonies were obtained, plasmid DNA was isolated from them and the presence of the TAp63 gene in correct orientation in respective vectors was confirmed by digestion with NotI (New England Biolabs, Whitby, ON, Canada).

**Densitometric Analysis of Western Blots**

Films were scanned, and densitometric analysis of the resulting digital images was performed using Quantity One software (Bio-Rad).
