# Supplementary figures and images for: ErbB2-driven downregulation of the transcription factor Irf6 in breast epithelial cells is required for their 3D growth
Source: Breast Cancer Res. 2018 Dec 13;20:151. doi: 10.1186/s13058-018-1080-1 (PMC6293553; doi:10.1186/s13058-018-1080-1)

## Slide 1
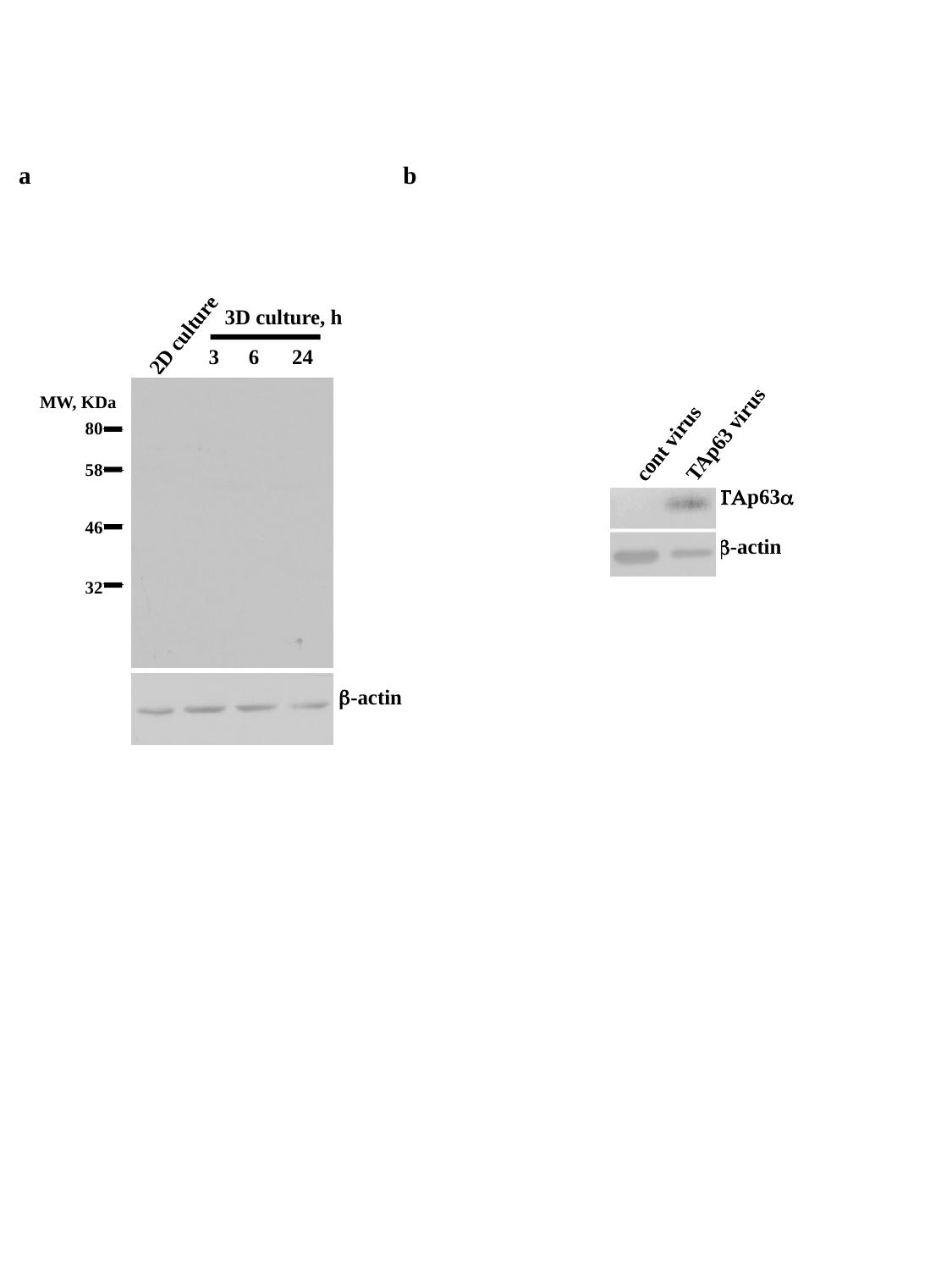

a
b
3D culture, h
2D culture
3
6
24
MW, KDa
80
58
46
32
TAp63 virus
cont virus
p63α
β-actin
β-actin

Supplement: Supplementary file 2 — Figure S1. TAp63 is not detectable in MCF10A cells. a MCF10A cells were cultured attached to (2D culture) or detached from (3D culture) the ECM for the indicated times and assayed for TAp63 levels by Western blotting by use of a TAp63-specific antibody. b To ensure that the TAp63-specific antibody was capable of recognizing TAp63 in our experimental conditions, we infected MCF-ErbB2 cells with a control or a TAp63a-encoding retroviruses. TAp63 levels in the cells were assayed by Western blotting using the indicated antibody. β-actin was used as a loading control. (PPT 188 kb) [file 13058_2018_1080_MOESM2_ESM.ppt]

## Slide 1
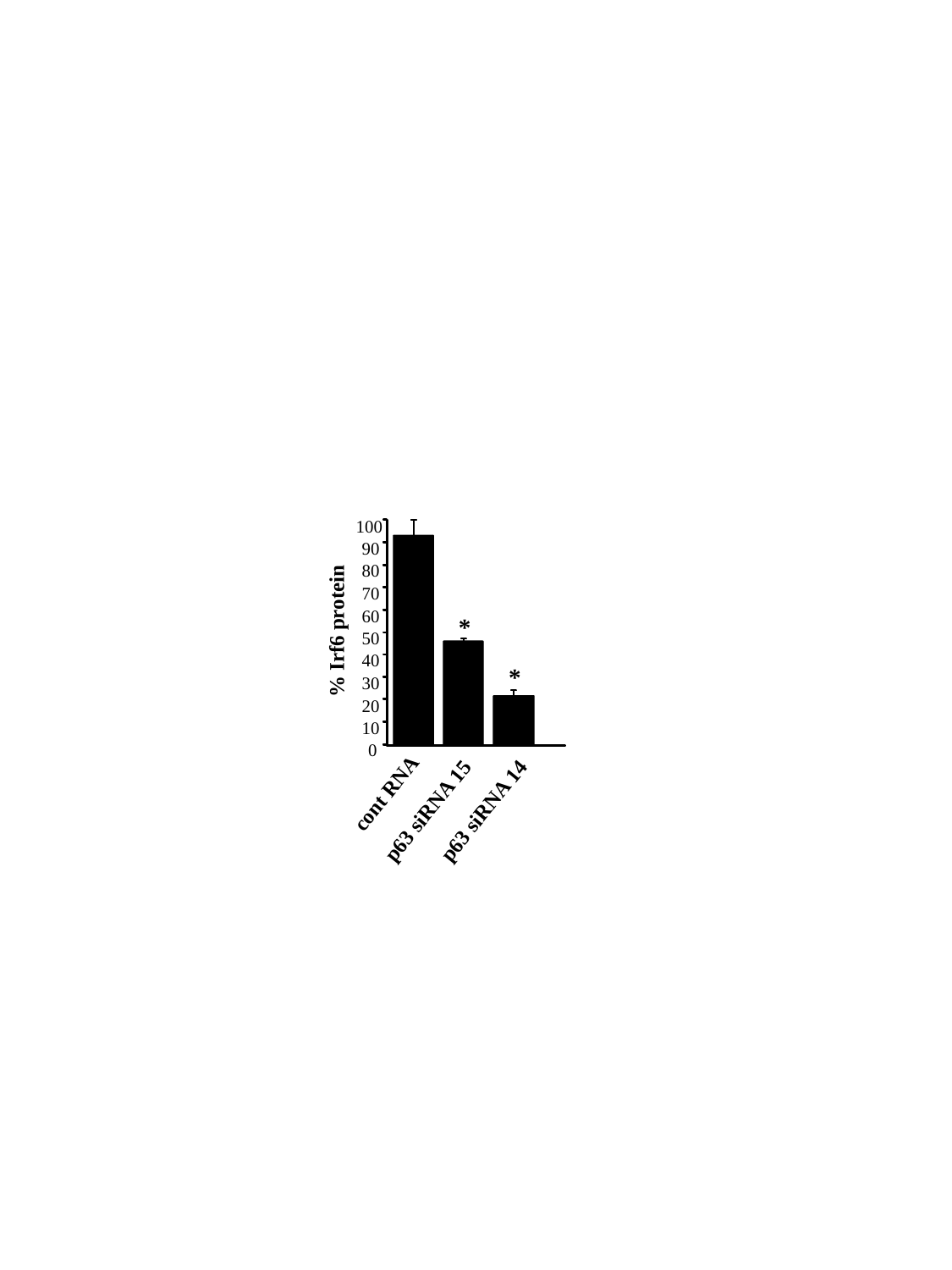

100
90
80
70
60
50
40
30
20
10
0
*
% Irf6 protein
*
cont RNA
p63 siRNA 15
p63 siRNA 14

Supplement: Supplementary file 3 — Figure S2. p63-specific siRNAs downregulate Irf6 in MCF10A cells in 3D culture. MCF10A cells transfected with 100 nM control RNA (cRNA) or p63-specific siRNA (p63siRNA) 14 or 15 were kept in 3D culture for 3 h and assayed for Irf6 expression by Western blotting. β-actin was used as a loading control in one experiment, and α-tubulin was used as a loading control in another independent experiment. Films were scanned, and densitometric analysis of the resulting digital images was performed. Irf6 protein levels were normalized to those of the loading controls. The data represent the average of two independent experiments plus the SD. * p < 0.05. (PPT 53 kb) [file 13058_2018_1080_MOESM3_ESM.ppt]
