# Supplementary material for: ErbB2-driven downregulation of the transcription factor Irf6 in breast epithelial cells is required for their 3D growth
Source: Breast Cancer Res. 2018 Dec 13;20:151. doi: 10.1186/s13058-018-1080-1 (PMC6293553; doi:10.1186/s13058-018-1080-1)
Supplement: Supplementary file 4 — Figure S3. ErbbB2 and Mek downregulate ΔNp63α in detached breast epithelial cells. MCF10A and MCF-ErbB2 cells (a–c) or MCF10A and MCF-MekDD cells (b–d) were cultured detached from the ECM (3D culture) for the indicated times and assayed for ΔNp63 levels by Western blotting. β-actin was used as a loading control. Fragments of panels a and d showing ΔNp63 levels are displayed in Fig. 6a and b, respectively. (PPT 318 kb) [file 13058_2018_1080_MOESM4_ESM.ppt]

## Slide 1
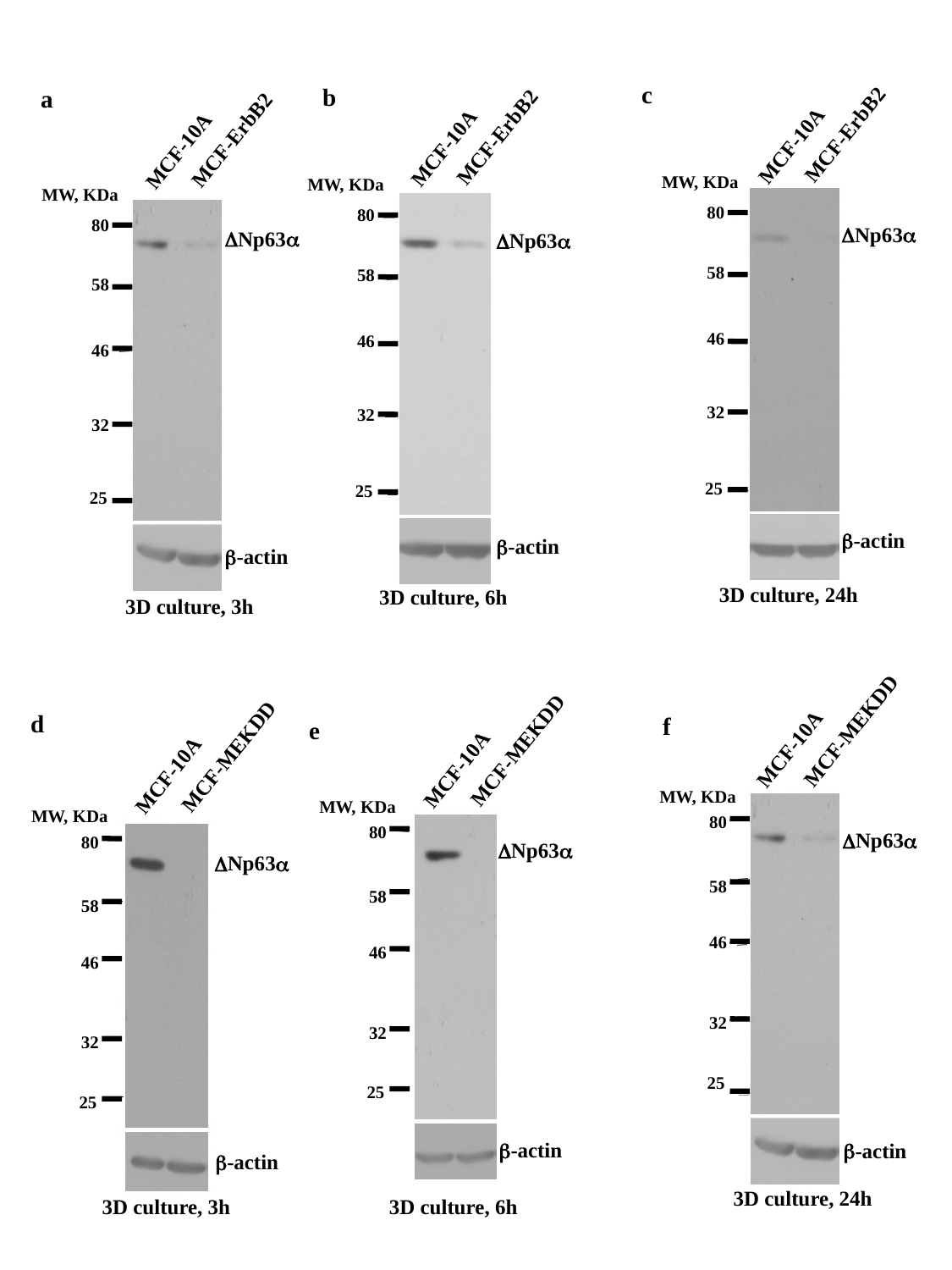

c
b
a
MCF-ErbB2
MCF-ErbB2
MCF-ErbB2
MCF-10A
MCF-10A
MCF-10A
MW, KDa
MW, KDa
MW, KDa
80
80
80
ΔNp63α
ΔNp63α
ΔNp63α
58
58
58
46
46
46
32
32
32
25
25
25
β-actin
β-actin
β-actin
3D culture, 24h
3D culture, 6h
3D culture, 3h
d
f
e
MCF-MEKDD
MCF-10A
MCF-MEKDD
MCF-MEKDD
MCF-10A
MCF-10A
MW, KDa
MW, KDa
MW, KDa
80
80
ΔNp63α
80
ΔNp63α
ΔNp63α
58
58
58
46
46
46
32
32
32
25
25
25
β-actin
β-actin
β-actin
3D culture, 24h
3D culture, 3h
3D culture, 6h
