# Supplementary material for: ErbB2-driven downregulation of the transcription factor Irf6 in breast epithelial cells is required for their 3D growth
Source: Breast Cancer Res. 2018 Dec 13;20:151. doi: 10.1186/s13058-018-1080-1 (PMC6293553; doi:10.1186/s13058-018-1080-1)
Supplement: Supplementary file 5 — Figure S4. Irf6 is upregulated in breast tumor cells after neoadjuvant trastuzumab-based therapy. Formalin-fixed, paraffin-embedded tumor sections obtained from patient 4 before (a, c) and after (b, d) the therapy were stained with an anti-Irf6 antibody. The samples were stained with hematoxylin (blue) and eosin (red) (H&E) (a, b) or with an anti-Irf6 antibody (brown) (d, e) and counterstained with hematoxylin (blue). (PPT 1479 kb) [file 13058_2018_1080_MOESM5_ESM.ppt]

## Slide 1
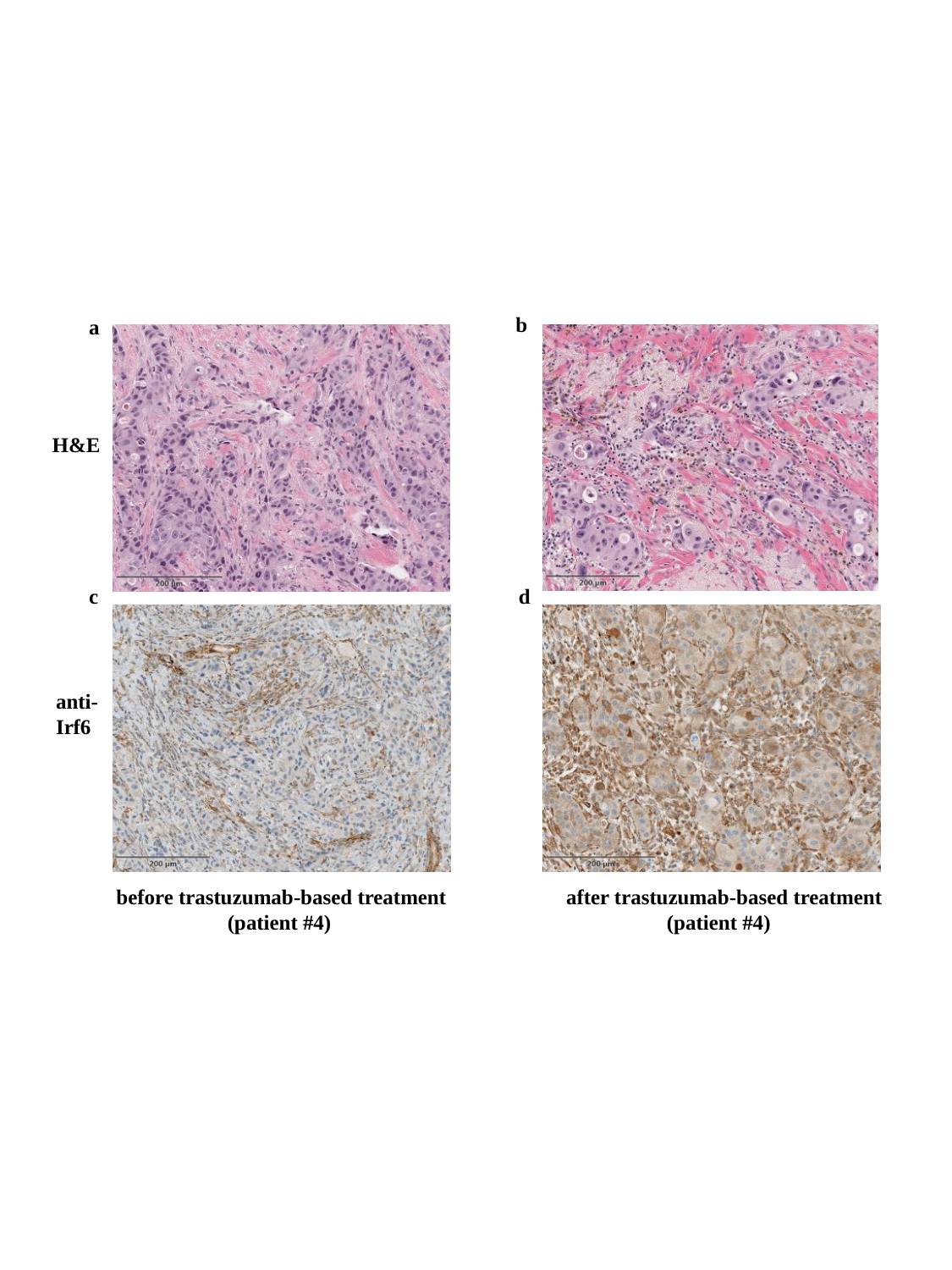

b
a
H&E
c
d
anti-
Irf6
before trastuzumab-based treatment
 (patient #4)
after trastuzumab-based treatment
 (patient #4)
